# Numerical Proportion Representation: A Neurocomputational Account

**DOI:** 10.3389/fnhum.2017.00412

**Published:** 2017-08-14

**Authors:** Qi Chen, Tom Verguts

**Affiliations:** ^1^School of Psychology, South China Normal University Guangzhou, China; ^2^Center for Studies of Psychological Application, South China Normal University Guangzhou, China; ^3^Guangdong Key Laboratory of Mental Health and Cognitive Science, South China Normal University Guangzhou, China; ^4^Department of Experimental Psychology, Ghent University Ghent, Belgium

**Keywords:** numerical cognition, numerical proportion representation, computational modeling, gain-field model, invariance and generalization

## Abstract

Proportion representation is an emerging subdomain in numerical cognition. However, its nature and its correlation with simple number representation remain elusive, especially at the theoretical level. To fill this gap, we propose a gain-field model of proportion representation to shed light on the neural and computational basis of proportion representation. The model is based on two well-supported neuroscientific findings. The first, gain modulation, is a general mechanism for information integration in the brain; the second relevant finding is how simple quantity is neurally represented. Based on these principles, the model accounts for recent relevant proportion representation data at both behavioral and neural levels. The model further addresses two key computational problems for the cognitive processing of proportions: invariance and generalization. Finally, the model provides pointers for future empirical testing.

## Introduction

Numerical processing is an important cognitive capacity across a variety of animal species. Accordingly, it is a topic of great interest in recent cognitive neuroscience and psychology, studied with methodologies as diverse as single-cell recording (Nieder and Miller, 2004), neuroimaging (Piazza et al., 2004), and developmental paradigms (Xu et al., 2005). Many studies focus on simple (i.e., single-variable) quantity representation, for example the number of objects in a set (Nieder and Miller, 2003) or the length of a line (Tudusciuc and Nieder, 2007). However, ratios of quantities (proportions, where two variables are combined) are an important and emerging field of study (Kallai and Tzelgov, 2009; Ganor-Stern et al., 2011; Holmin and Norman, 2012; Siegler et al., 2013). One recurring finding here is that the classical distance effect from numerical cognition (Moyer and Landauer, 1967) is also robustly observed for proportions (Schneider and Siegler, 2010). Another relevant finding is that this distance effect tends to be approximately symmetrical (Vallentin and Nieder, 2008). However, the mechanistic interpretation of these data has remained unclear. Computational model can help us integrate these data in a computational framework and make novel experimental predictions. Unfortunately, there are as yet no computational proposals on how such ratios are processed. Such a proposal is the content of the current paper.

On a computational level, learning and representing proportions (either non-symbolic or symbolic) involve two core computational properties. The first is the *invariance property* (Salinas and Abbott, 1997). This means that both humans and non-human animals can represent abstract length proportions, and ignore the exact length of two lines. For example, they can represent the proportion 1:4 for different combinations of line length (e.g., 1 cm vs. 4 cm and 2 cm vs. 8 cm). Using fMRI adaptation paradigm with healthy subjects, Jacob and Nieder (2009) found clear evidence to support this invariance property of proportion representation. The second is the *generalization property*. This means that after learning, both humans and non-human animals can generalize learned proportions to novel proportions. In a recent study (Vallentin and Nieder, 2008), macaques were trained on length proportions between two lines (1:4, 2:4, 3:4, and 4:4). Remarkably, precision was the same for the trained proportions (e.g., 1:4, 2:4, 3:4, and 4:4) and transfer proportions (e.g., 3:8 and 5:8). As noted by the authors of that study, this suggests that the animals had a conceptual understanding of abstract proportions.

A series of recent studies (for review, see Jacob et al., 2012; Siegler et al., 2013) begin to shed light on the neural basis of proportion representation and these two computational problems. To investigate the neuronal code of proportions, Vallentin and Nieder (2008, 2010) recorded electrophysiological data from cells in the frontal and parietal cortex of behaving rhesus monkeys in a delayed match-to-sample task, in which monkeys matched sample and test proportions, defined by the ratio of the length of two lines. Approximately, 30% of the prefrontal cortex (PFC) neurons and approximately 16% of inferior parietal cortex neurons encoded one of the trained proportions. These neurons code magnitude proportion information with unimodal tuning curves, characterized by a maximum firing rate for a specific proportion and decreasing as the distance from this preferred ratio increased. This coding mechanism, called place code (Verguts and Fias, 2004) or labeled line code (Nieder and Merten, 2007) is also used for simple magnitude representation (Nieder and Miller, 2003).

Whereas there are several computational models about simple quantity representation (Dehaene and Changeux, 1993; Grossberg and Repin, 2003; Stoianov and Zorzi, 2012), no attempts have been made to exploring ratios of quantities (proportions and fractions) computationally. To fill this gap, here we propose a gain-field model for proportions. This model is based on two recent neuroscientific findings. The first is gain modulation, which is a ubiquitous mechanism for information integration in the mammalian brain (Salinas and Bentley, 2009). Salinas and colleagues (Salinas and Thier, 2000; Abbott, 2005) have pointed out that different types of information can be integrated by multiplicative gain modulation, which is implemented by radial basis function neurons, at the neural level. A well-known example is that different spatial representations are mapped on radial basis function neurons in parietal cortex, and transformation on the original spatial representations is implemented by projections from the radial basis function neurons to different spatial representations. This theory is supported by much neurophysiological evidence (Pouget and Snyder, 2000). An example is the transformation from visual information in eye-centered coordinates to head-centered coordinates, which is useful for correctly turning the head toward a seen object. For this spatial transformation, the response profile of radial basis function neurons in parietal cortex can be modeled by a product of retinotopic position and eye position receptive fields (Pouget and Sejnowski, 1997). Modeling studies have shown that gain modulation can also support many other cognitive tasks, including: arbitrary sensory-motor remapping (Salinas, 2004), generation of motor sequences (Salinas, 2009), serial order representation (Botvinick and Watanabe, 2007), and elementary arithmetic (Chen and Verguts, 2012). In this study, we apply this well-motivated and ubiquitous framework to proportion representation.

The second relevant neuroscientific finding on which our model is based, is how simple quantity is represented. Recent studies in non-human animals and humans using diverse methodologies have provided detailed answers to this problem (for review, see Nieder and Dehaene, 2009). Representation of simple quantity is instantiated by a distinct set of place coding neurons in regions of the prefrontal and posterior lobes, each tuned maximally to a specific number, with approximately Gaussian tuning curves when plotted on a logarithmic axis (Nieder and Miller, 2003). Importantly, neuronal population coding of continuous quantity (line length) in the primate posterior parietal cortex uses the same coding mechanism (Tudusciuc and Nieder, 2007).

In the present study, we integrate these two findings into a model for proportion representation. In the following, we first describe the model in detail, then report a series of simulation studies, and conclude by a General Discussion.

## Materials and Methods

### Network Architecture

**FIGURE 1:**
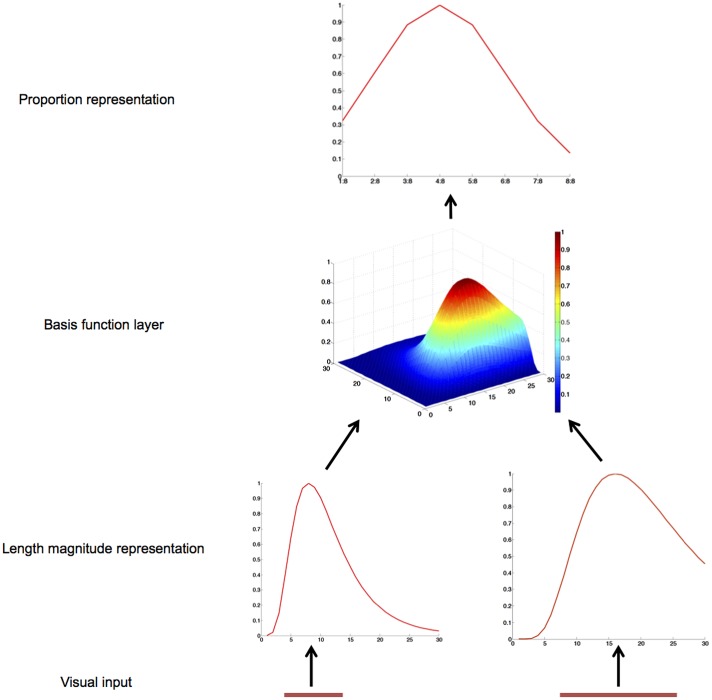
Schematic diagram and operation of the gain-field model of proportion representation. The two operands of a proportion problem, the length of two lines, are mapped onto two length magnitude representation layers. The basis function layer combines these two inputs and sends activation to the proportion representation layer.

The model architecture is shown in **Figure 1**. Its core is a three-layer feedforward structure, consisting of 60 input, 900 hidden, and 8 output neurons.

There are two groups of 30 input neurons, one for the length of each line (Vallentin and Nieder, 2008). Presentation of a line to such an input layer results in a logarithmically compressed Gaussian activation curve (Tudusciuc and Nieder, 2007). In particular, each input neuron is maximally activated by a preferred length, *p*, and the activation value of each input neuron is based on the logarithmic distance between this preferred length and the actual length, *s*, according to a Gaussian function (see **Figure 1**):

(1) $$\begin{array}{c} R_{p}(s)=\exp(-\frac{{\left(\ln s-\ln p\right)}^{2}}{2\sigma^{2}}) \end{array}$$

where R_p_(s) is the activation of the neuron with preferred length *p* for a target length *s* (Botvinick and Watanabe, 2007). Note that lns - lnp = ln(s/p), so even at the simple number representation level a ratio is calculated, although its calculation is different from neurons in the hidden layer.

Each neuron in the hidden layer receives input from a unique combination of one neuron from each of the two layers of input neurons, so the hidden layer comprises 900 basis function neurons. The activation value of a hidden layer neuron equals the product of its input neurons’ activation values:

(2) $$\begin{array}{c} H_{j}{=R}_{x}{(s)R}_{y}(s) \end{array}$$

where H_j_ is the activation of the hidden unit receiving input from two input neurons from the two groups, with preferred length *x* and *y*, respectively.

To simulate the target data from Vallentin and Nieder (2008) where eight proportions were used, our output layer also has eight neurons. At this output layer, there is one neuron for each proportion (1:8, 2:8, 3:8, 4:8, 5:8, 6:8, 7:8, and 8:8). Every output neuron receives inputs from all hidden neurons as follows:

(3) $$\begin{array}{c} O_{i}=\sum_{j}w_{ij}H_{j} \end{array}$$

where w_ij_ represents the synaptic connection weight from hidden neuron *j* to output neuron *i.*

### Simulation

**FIGURE 2:**
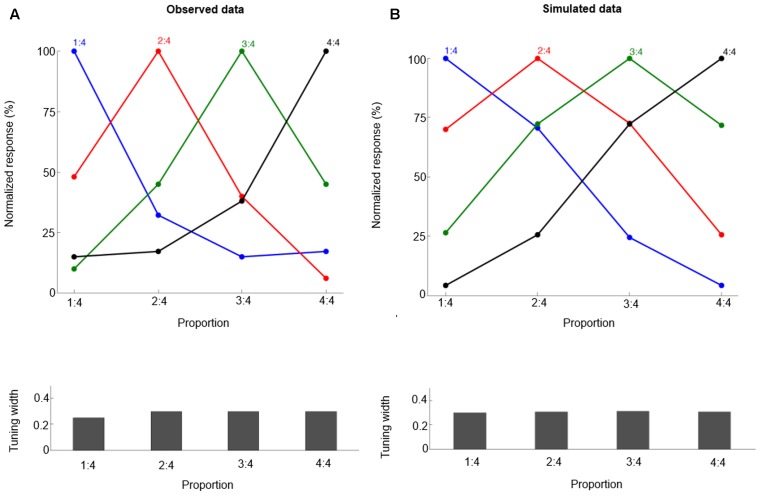
The neural tuning curve properties for proportions 1:4, 2:4, 3:4, and 4:4. Each curve is for neurons tuned to a specific proportion, and the labels on the X-axis are for specific proportions that are presented. Observed data (**A**, from Vallentin and Nieder, 2008) and simulated data **(B)**. The normalized tuning functions are plotted relative to the preferred proportion. Bottom panels show the standard deviation values (Tuning width) for each tuning curve.

The weights between the hidden layer and the output layer are trained such that they minimize the average squared error between intended and actual responses. These weights are initially set to random values. Input pairs with corresponding targets are presented in random order, and after each training trial the weights are updated by the delta learning rule:

(4) $$\begin{array}{c} \Delta w_{ij}=\sigma (T_{i}-O_{i})H_{j} \end{array}$$

where α is the learning rate, and T_i_ is the target value for output neuron *i*. We define the target *T* as a Gaussian function curve across the output layer (with standard deviation 0.3). One motivation for this target setting is the fact that the neuronal population tuning curve can be fitted with a Gaussian function with standard deviation about 0.3 (reported in Vallentin and Nieder, 2008; **Figure 2A**). See a recent study (Weisswange et al., 2011) for a similar approach in their model of Bayesian cue integration and causal inference. To mimic the setting of Vallentin and Nieder (2008), four proportions are trained: 1:4, 2:4, 3:4, and 4:4. Each is specified by three concrete training examples (different line length combinations) so there are 12 training pairs. The learning rate is 0.01 for all simulations. The model was tested 10 times, with 20000 training trials in each replication. The results are averaged across the 10 replications.

We follow the method of Vallentin and Nieder (2008) and fit the neuronal population tuning curve with a Gaussian function. For all proportions, the goodness of fit (r^2^) of the Gaussian function is determined, and the tuning curves’ standard deviation value (the half-bandwidth of the fitted Gaussian function) is derived.

**FIGURE 3:**
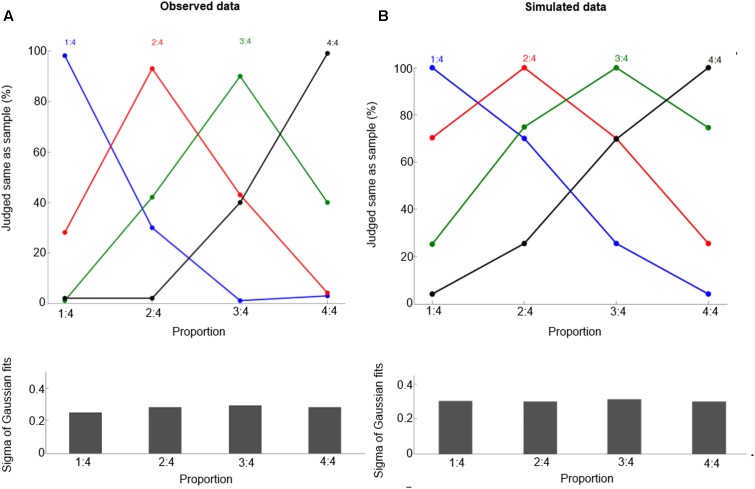
Behavioral performance for proportions 1:4, 2:4, 3:4, and 4:4. Observed data (**A**, from Vallentin and Nieder, 2008) and simulated data **(B)**. These curves show the percentage of trials in which one judged sample and test displays containing the same proportion, and the curves refer to the preferred proportions. Bottom panels show half bandwidth (Sigma of Gaussian fits) of the Gaussian functions fitted to behavioral performance curves.

To test the model’s performance for the delayed match-to-sample task, we transform the neuronal population response (**Figure 2B**) into behavioral performance (**Figure 3B**). The macaques are trained to perform a delayed match-to-sample task, in which they matched sample and test proportions, defined by the ratio of the length of two lines, in order to obtain reward. For simplicity, here we use an intuitive way to calculate the probability of responding ‘same’ [for a more elaborate and complex expression based on Signal Detection Theory and Bayesian decision, see (Dehaene, 2007); for a more detailed neural model, see Engel and Wang, 2011]. It is given by

(5) $$\begin{array}{c} P_{same}(n_{1},n_{2})=\frac{o_{n_{2}(n_{1})}}{o_{n_{1}(n_{1})}} \end{array}$$

where o_n_2_(n_1_)_ is the activation of the neuron with preferred proportion n_2_ when a proportion n_1_ and o_n_1_(n_1_)_ is the activation of the neuron with preferred proportion n_1_ when a proportion n_1_. Furthermore, we use this equation for calculating the probability of responding ‘same,’ and evaluate the goodness of fit of a Gaussian function. The goodness (r^2^) is determined, and the standard deviation (the half-bandwidth of the fitted Gaussian function) is derived. Note that Eq. (5) is just the simplest way to create a probability P_same_(n_1_,n_2_) that is a monotonic function of the distance |n_1_ - n_2_| based on the model output; future work should also consider whether the model can capture trial-to-trial variability in this task.

## Results

After training, our model can produce correct proportion representation. Representative simulated neural tuning curves are shown in **Figure 2B**. The goodness of fit of a Gaussian function is approximately 1. These neurons code proportion information with unimodal tuning curves, characterized by a maximum firing rate for a specific proportion independent of the particular numbers making up the proportion (invariance property). This is consistent with single-cell data (**Figure 2A**, Vallentin and Nieder, 2008). The simulated tuning curves’ standard deviation (tuning width) is about 0.3. The tuning width changes with different ratios, although this effect is subtle (as in the empirical data). One departure from observed data is that in the simulation there is no clearly narrower tuning for 1:4 than for the other proportions. Also the simulated behavioral performance is comparable to macaques’ performance (compare **Figures 3A** and **3B**, the mean goodness of fit of a Gaussian function is again approximately 1). Furthermore, our model clearly shows a distance effect (see **Figure 3B**). For example, the percentage of trials in which 2:4 is judged the same as sample 3:4 is higher than the percentage of trials in which 1:4 is judged the same as sample 3:4.

**FIGURE 4:**
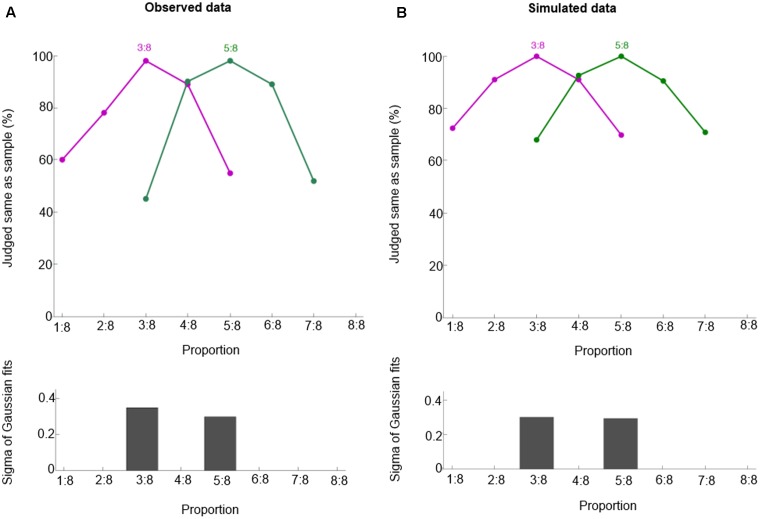
Behavioral performance for proportions 3:8 and 5:8. Observed data (**A**, from Vallentin and Nieder, 2008) and simulated data **(B)**. These curves show the percentage of trials in which one judged sample and test displays containing the same proportion. Bottom panels show half bandwidth of the Gaussian functions fitted to behavioral performance curves.

To check whether our model can generalize from learned to novel proportions, we test our model on 3:8 and 5:8. Like macaques (**Figure 4A**), our model can respond appropriately to 3:8 and 5:8, as shown in **Figure 4B** (mean r^2^ = 0.99). Thus, our model is able to generalize based on its basis function layer and place coding representation. These two properties entail that similar representations are trained similarly, so training target proportions (e.g., 1:4, 2:4, 3:4, and 4:4) implicitly also trains similar proportions (e.g., 3:8 and 5:8).

## Discussion

Based on two well-evidenced neuroscientific findings (gain modulation coding and neural coding of simple number), we proposed a novel model to account for recent findings of proportion representation. The model also provides a minimal computational framework to solve two key computational problems for proportion representation: invariance and generalization. Our model is of the connectionist variety. The connectionist approach is to model cognition based on the idea that the knowledge underlying cognitive activity is stored in the connections (weights) among neurons (McClelland et al., 2010). In particular, we used the basis function framework, in which input-to-hidden connections are fixed, and hidden units respond to a restricted part of the two input spaces. This allows constructing a powerful model, in combination with a simple (hidden-to-output, delta) learning rule. We have used this computational framework in earlier work to model elementary arithmetic (Chen and Verguts, 2012), where two numerical representations are combined together. The current study can thus be considered an extension of our previous work (Chen and Verguts, 2012). The study is consistent with our general approach to instantiate core computational principles as simply as possible, thus to investigate which principles are sufficient to account for numerical cognition.

One possible departure from neurobiology is that the hidden layer was much bigger than the input layers. However, this simplification is well-motivated. First, the current input layer only represents a simplified approximation to the actual input. Second, in reality the hidden layer probably approximates the required input-output function with a less extensive set of basis function (Botvinick and Watanabe, 2007). We performed additional simulations with less basis functions. This led to very similar results.

The model can help addressing two core questions about proportion representation. The first is how the analog code for proportions is constructed (Jacob et al., 2012). According to our model, individual components of a proportion (for example, the length of two lines) are mapped onto two length magnitude representation layers. The basis function layer combines these two inputs and sends activation to the proportion representation layer. In this sense, proportions are represented at the apex of the processing hierarchy; different neurons encode either simple magnitude or proportions separately, and these two kinds of neurons are linked by basis function neurons. Both simple magnitude and proportion neurons have been found in the same cortical regions [e.g., bilaterally in the intraparietal sulcus (IPS) and lateral PFC (Vallentin and Nieder, 2008, 2010)]. The model predicts that these basis function neurons for proportion representations can also be found in the same cortical regions. Such basis function neurons would be maximally tuned to a specific combination of line lengths, with approximately two-dimension Gaussian tuning curves when plotted on a logarithmic axis (see Basis function layer in **Figure 1**). Furthermore, the model also predicts that there are both invariant and variant proportion cells. On the one hand, in the proportion representation layer, cells respond to any pair with one specific proportion (e.g., 1:2). On the other hand, in the basis function layer, cells only respond to specific pairs (e.g., 2 cm: 4 cm, only for a particular combination of line length). In their data analysis, Vallentin and Nieder (2008) only included invariant cells. However, there is some suggestion that variant neurons occur in the data. Indeed a substantial number of neurons (16%) show a proportion × protocol interaction (Vallentin and Nieder, 2008), suggesting that they selectively respond to some quantitative combinations (e.g., 1:2 with quantities 1 and 2) but not others (e.g., 1:2 with quantities 2 and 4). A followup investigation or reanalysis of the data from (Vallentin and Nieder, 2008) may reveal whether such variant cells (basis function neurons) indeed exist and whether (as we predict) the variant cells are activated slightly earlier than the invariant ones.

The second core question concerns the componential vs. analog (holistic) representation of proportions. Single-cell recording from macaques (Vallentin and Nieder, 2008, 2010) and neuroimaging studies with human adults using the adaptation paradigm (Jacob and Nieder, 2009) strongly favor holistic (place coding) representations of proportion. In contrast, a lot of behavioral studies with human participants and symbolic proportions (i.e., fraction) clearly support componential representation (Meert et al., 2010). Our model provides a unified explanation for both componential and holistic representation of proportion: the simple magnitude representations (**Figure 1**) can be considered as the neural base for componential representation, and place coding of proportion representation in the output layer as neural base for holistic representation. In this sense, our model implies that componential and holistic representations occur simultaneously.

Until now, we applied our computational model to continuous proportions. However, the same computational model principle and structure can be applied to discrete ratios (e.g., ratio of two vs. five balls). In our opinion, the difference between continuous and discrete ratio architectures resides in the input (to the model). For continuous ratios, the input layer represents continuous quantities (e.g., length); for discrete ratios, the input layer represents discrete quantities (number of balls in the example). Furthermore, our model can be generalized to explain symbolic proportions (fractions). Again, the concept of fractions encounters the same key computational problems of invariance and generalization, and can be addressed by a similar model with different input layers. This can partly explain why it is more difficult to deal with fractions than natural numbers for children (Siegler et al., 2011, 2013). To correctly name and deal with fractions, the numerator and denominator need to be combined (Jacob et al., 2012).

Our model resembles exemplar models of categorization (Kruschke, 1992; Love et al., 2004) and visual object recognition (Riesenhuber and Poggio, 1999, 2000). In particular, the hidden layer combines and integrate different pieces of information in a non-linear manner and sends activation to the output layer (e.g., category or object representation layer). This similarity is no coincidence because categorization and visual object recognition encounter very similar challenges of invariance and generalization, both of which are very well-handled by the basis function model architecture.

## Conclusion

We propose that a gain-field model accounts for extant data concerned with proportion representation. This theory has several advantages: it is computationally implemented, its neural underpinnings are beginning to be known (Jacob et al., 2012), and it provides some testable predictions.

## Author Contributions

QC and TV developed the study concept and contributed to the study design; computer simulation was performed by QC; the data analysis and interpretation was performed by QC and TV; QC and TV wrote the manuscript.

## Conflict of Interest Statement

The authors declare that the research was conducted in the absence of any commercial or financial relationships that could be construed as a potential conflict of interest.
